# The Impact of Angle Lambda on Patient Satisfaction after Optiflex Trio Trifocal Intraocular Lens Implantation

**DOI:** 10.1155/2023/7911449

**Published:** 2023-06-17

**Authors:** Sultan Kaya Ünsal, Ertan Sunay

**Affiliations:** Veni Vidi Eye Hospital, Istanbul 34728, Turkey

## Abstract

**Purpose:**

To evaluate the vision-related quality of life (QOL), visual acuities, and refractive outcomes of patients with different angle lambda (*λ*) after a trifocal intraocular lens (IOL) implantation at Veni Vidi Eye Hospital, İstanbul, Turkey.

**Methods:**

This retrospective, nonrandomized, and noncomparative case series included patients who had phacoemulsification bilaterally with the implantation of a trifocal IOL (Optiflex Trio) and responded to a vision-related QOL questionnaire measuring patient satisfaction. The patients were divided into two groups according to the angle *λ* with a cutoff value of 0.5 mm. Evaluations were conducted to examine the monocular visual acuities, spherical equivalents, corneal astigmatism measured 3 months after surgery, and outcomes of the QOL questionnaire in the two groups.

**Results:**

The study examined 130 eyes from 65 patients aged from 41 to 78 years old. There were no statistically significant differences between the two groups at 3 months after surgery in terms of uncorrected distance visual acuity (UDVA), monocular uncorrected intermediate visual acuity (UIVA), monocular uncorrected near visual acuity (UNVA), spherical equivalent, and corneal astigmatism (*P* > 0.05). Patients with a greater angle *λ* had significantly more difficulty going out to see movies, plays, or sporting events (*P*=0.02), driving at night (*P*=0.002), and driving in difficult conditions (*P* < 0.001) than patients with a lower angle *λ*.

**Conclusions:**

The Optiflex Trio showed good results in terms of visual acuity at all distances, positive refractive outcomes, and high patient satisfaction in daily life according to the QOL questionnaire. An angle *λ* greater than 0.5 mm may potentially cause dysphotopsia symptoms, especially during nighttime activities.

## 1. Introduction

With recent innovations in intraocular lens (IOL) production, IOL implantation has become a refractive procedure [1–3]. The addition of a new focal point for intermediate distance in trifocal IOLs has reduced the limitations of multifocal IOLs [4]. Visual quality can be improved significantly through the implantation of trifocal IOLs, but some patients with higher visual acuity values might feel disadvantaged in their daily activities. Even with excellent uncorrected visual acuity (UCVA), some patients are dissatisfied due to dysphotopsias such as glare, halos, and starbursts.

According to previous studies on IOL, causes of the dysphotopsia include misalignment, posterior capsular opacification, retained lens fragments, postoperative astigmatism, and dry eye syndrome [5, 6]. Furthermore, postoperative higher-order aberrations can result from large deviations between the visual axis, optical center, and pupillary axis of the IOL, which may decrease vision quality [7]. The angle lambda (*λ*) has also been reported as an important factor in aberrations in the human eye [8].

In this study, we used the Visual Function Questionnaire-25 (VF-25) of the National Eye Institute (NEI) [9] to measure the effect of the angle *λ* on the satisfaction of patients with bilateral trifocal IOL implantation.

## 2. Methods

### 2.1. Patients

This retrospective nonrandomized study included patients who underwent cataract surgery with implantation of Optiflex Trio IOLs (Optiflex Trio, Biotech Europe Meditech Inc.) between March 2019 and January 2020 at the Veni Vidi Eye Hospital in İstanbul, Turkey. The study was approved by the Ethics Committee of Marmara University, and written informed consent was obtained from each patient. The study was also conducted in accordance with the principles outlined in the Declaration of Helsinki.

The included patients had age greater than 40 years, age-related cataract, and corneal astigmatism less than 1.00 diopter (D). Patients were excluded if they had preoperative ocular diseases such as dry eye syndrome, glaucoma, retinal pathologies, corneal diseases, uveitis and unexpected postoperative astigmatism or irregular corneal astigmatism greater than 1.00 D, IOL decentration, previous ocular surgery, postoperative, or intraoperative complications.

### 2.2. Preoperative Examination

All pre- and postoperative ophthalmologic examinations were performed by the same ophthalmologist (KAYA UNSAL, S). The preoperative examination included anterior segment evaluation with slit-lamb examination, measurement of intraocular pressure by a noncontact tonometer (TOPCON CT-80; Topcon Medical Systems), retina evaluation using 90 D lens examination after pupil dilation, and measurement of the monocular corrected and uncorrected distance visual acuities (UDVAs) under photopic light conditions using Snellen visual charts.

**Figure 1:**
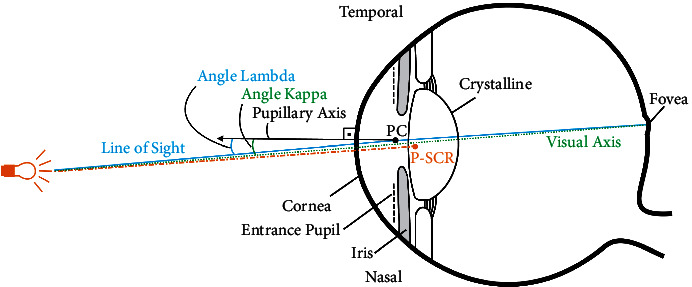
Diagram of angle kappa, angle lambda, line of sight, pupillary axis, visual axis, pupillary center (PC), and Purkinje–Sanson corneal reflex (P-SCR).

Corneal topography analysis was performed and included measurement of the angle *λ* (Sirius; Costruzione Strumenti Oftalmici). The angle *λ* is the angle between the line of sight and the pupillary axis [10, 11]. The pupillary axis is the line perpendicular to cornea passing through the center of the pupil's entrance, as demonstrated in Figure 1. However, the actual angle *λ* value is not measured by topography devices. The distance of the corneal vertex to the pupillary axis is used as an approximation of the angle *λ* and reported as the *λ* intercept in topography data. IOL power was calculated using optical biometry (LENSTAR; Haag-Streit) and the SRKT formula. All cases were targeted for emmetropia.

### 2.3. Intraocular Lens

The Optiflex Trio is a single diffractive-refractive, 360°, square-edge, aspheric, and trifocal intraocular lens that is used for presbyopia correction. It is composed of hydrophobic acrylic containing natural chromophores. The IOL has an optic size of 6.00 mm and overall size of 13.00 mm. The refractive index of the IOL is 1.48. The Optiflex Trio has additions of +1.85 D for intermediate vision and +3.50 D for near vision, which are used with the aim of achieving optimal reading distances of 72 and 38 cm, respectively. The lens material and its natural yellow chromophores prevent the risk of age-related macular degeneration (ARMD), circadian rhythm disruption, and altered color perception.

### 2.4. Surgical Technique

Phacoemulsification (Centurion, Alcon Laboratories, Inc.) was used in all surgeries, which were performed by the same surgeon (KAYA UNSAL, S). A clear 2.8-mm corneal incision (right 180°, left 0°) and 2 side ports were created. The same viscoelastic was used in all surgeries to deepen the anterior chamber with 1.65% sodium hyaluronate and 4% sodium chondroitin sulfate (DiscoVisc, Ophthalmic Viscosurgical Device, Alcon Laboratories). After performing capsulorhexis, phacoemulsification was performed, and the IOL was implanted. Finally, all remaining ocular viscoelastic devices (OVDs) under the IOL were removed. The postoperative topical medicines were 0.5% moxifloxacin and 0.1% dexamethasone phosphate four times per day and 0.15% brimonidine tartrate twice per day for 1 month.

### 2.5. Postoperative Examination

A biomicroscopic examination was performed on the first day after surgery, and then the detailed postoperative assessments were performed at 1 and 3 months. In the examinations, measurements of monocular uncorrected intermediate (70 cm) and near (40 cm) visual acuities under photopic light conditions were performed in addition to preoperative examinations. At 3 months after the IOL implantation, the Turkish version of the NEI-VF-25 questionnaire was administered. The NEI-VF-25 is easy to implement and helps to improve patient compliance. The questionnaire was developed by Magnione et al. [12] and uses a five-point grading scale to measure patients' performance in daily activities.

### 2.6. Statistical Analysis

SPSS software (version 26.0, IBM Corp.) was used for statistical analyses. Variables are reported as the mean ± standard deviation (SD). The Kolmogorov–Smirnov test was used to check the compatibility of the data with a normal distribution. The differences between *λ* groups were checked with a Student's *t*-test, which was used after log transformation of the original data if it was not normally distributed. The chi-squared test was used to examine the statistical differences between categorical variables. The *P* value was considered as 0.05 to assess statistical significance.

## 3. Results

130 eyes from 65 patients aged from 41 to 78 years old were included in this study. There were 37 women and 28 men, and the mean age was 57.09 ± 7.08 (SD) years. All of the patients with regular cataract surgeries were divided into 2 groups based on their angle *λ* distance. The group with lower *λ* (0 < *λ* < 0.5 mm) comprised 49 eyes, and that with higher *λ* (*λ* ≥ 0.5 mm) comprised 81 eyes.

### 3.1. Preoperative Measurements

No statistically significant differences were found in the preoperative measurements of the two groups.  Table 1 shows the preoperative measurements of the patients according to group.

### 3.2. Postoperative Measurements

After 3 months, the UDVA was 0.10 ± 0.10 (median: 0.10), the monocular uncorrected intermediate visual acuity (UIVA) was 0.20 ± 0.08 (median: 0.20), and the monocular uncorrected near visual acuity (UNVA) was 0.17 ± 0.08 (median: 0.2). These results show that patients had good distant, intermediate, and near vision after trifocal IOL implantation. All postoperative parameters showed no significant differences between the two groups, including UDVA, UIVA, UNVA, spherical equivalent, and corneal astigmatism (*P* > 0.05). The mean postoperative visual acuities for all distances are shown in Table 2. None of the patients had severe dysphotopsia symptoms or required glasses for any activity, and none of them had significant posterior capsule opacification (PCO) requiring YAG laser capsulotomy.

### 3.3. Questionnaire Responses

The NEI-VF-25 quality of life (QOL) questionnaire was administered at the 3-month postoperative examination. The questionnaire includes three main parts: 4 questions related to overall health and vision, 12 questions related to difficulty in daily activities, 9 questions related to vision problems, and 11 subscale questions related to near vision, distant vision, etc.

Regarding the first part of the questionnaire, there were no significant differences between the two groups in general health (3.02 ± 0.48; 1: excellent; 5: poor) and overall vision (1.34 ± 0.52). The overall vision results after trifocal IOL implantation were very satisfactory.

**Figure 2:**
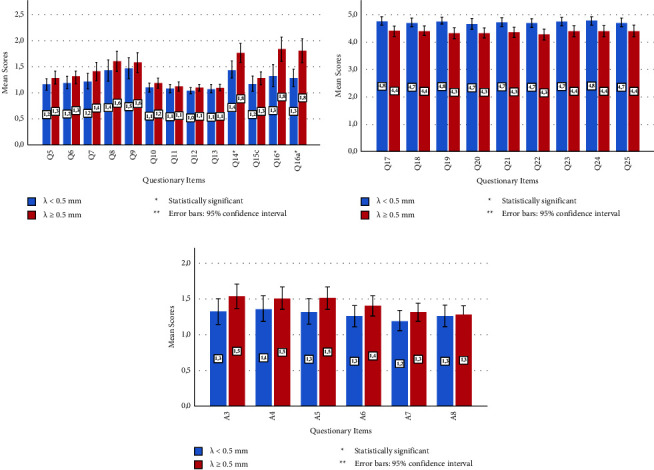
Outcomes of the NEI-VF-25 QOL questionnaire. (a) Activities: grading scale: 1, no difficulty at all; 2, a little difficulty; 3, moderate difficulty; 4, extreme difficulty; 5, stopped doing this because of your eyesight. (b) Response to vision problems: grading scale: 1, all of the time; 2, most of the time; 3, some of the time; 4, a little of the time; 5, none of the time. (c) Near and distance vision: grading scale: 1, no difficulty at all; 2, a little difficulty; 3, moderate difficulty; 4, extreme difficulty; 5, stopped doing this because of your eyesight.

Regarding the difficulty experienced by patients in daily activities, the most challenging were going out to see movies, (1.65 ± 0.76), driving at night (1.62 ± 0.86), and driving in difficult conditions (1.58 ± 0.82). For only these three activities, patients with a greater angle *λ* (≥0.5 mm) had significantly more difficulty than patients with a lower angle *λ* (<0.5 mm). There was no significant difference in other activities between these groups.  Figure 2(a) illustrates the patients' satisfaction levels with daily activities.

Concerning the responses to vision problems, the median satisfaction score of all patients was 5 for each question (the highest level), and there was no significant difference between groups, as shown in Figure 2(b).

Regarding the subscale questions, the mean and median scores of the questionnaire items related to near vision (A3, A4, and A5) were 1.46 ± 0.70 and median 1.00, while those for items related to distant vision (A6, A7, and A8) were 1.30 ± 0.57 and 1.00, respectively. These results indicate that the trifocal IOL implantation achieved very satisfactory patient outcomes for near and distant vision, as shown in Figure 2(c).

## 4. Discussion

Generally, visual acuity has been the main parameter examined in many studies measuring patient satisfaction after multifocal or trifocal IOL implantation. However, visual acuity alone is not sufficient to measure patient satisfaction. Photopic symptoms, contrast issues, and visual performance in daily activities must also be considered [4]. Some patients with higher visual acuity might still feel disadvantaged in their daily lives. One of the ways to measure this type of dissatisfaction is through QOL questionnaires.

Previous studies have evaluated the performance of trifocal IOLs, and the visual acuity and refractive outcomes of these studies are consistent with the outcomes of the present study [13–17]. Brar et al. [15] examined the refractive outcomes and visual acuity results after implantation of the Optiflex Trio, which is the same IOL as in this study. They examined 54 eyes from 27 patients, and binocular UDVA of 20/20 or better was achieved for 78% of patients. For 93% of eyes, SE refraction was between −0.50 and + 0.50 D, while refractive cylinder less than 0.50 D was achieved for 94% of eyes. The binocular visual acuity results at 12 months were reported as 0.01 ± 0.05 LogMAR for the mean UNVA and 0.07 ± 0.06 and 0.03 ± 0.05 LogMAR for the mean UIVA at 60 and 80 cm, respectively.

Although Brar et al. reported visual and refractive outcomes; no study has measured the impact of the Optiflex Trio on patient satisfaction and performance in daily activities using a validated methodology. Therefore, these aspects were evaluated using the NEI-VF-25 QOL questionnaire in this study.

The average values were between 1.00 and 2.00 for the questions related to activities and between 4.00 and 5.00 for the questions related to responses to vision problems. These averages indicate high patient satisfaction according to the NEI-VF-25 QOL questionnaire. The results show high vision-related QOL values and high patient satisfaction rates at 3 months after the implantation of the Optiflex Trio. Spectacle independence was obtained for all distances.

The activities with the highest difficulty were going out to see movies (1.65 ± 0.76), driving at night (1.62 ± 0.86), and driving in difficult conditions (1.58 ± 0.82). The higher difficulty levels during nighttime activities might have resulted from halo and glare symptoms under photic conditions with the trifocal IOL [4].

Kohnen et al. [16] reported that 93% of patients had dysphotopsia and double vision symptoms after implantation of a trifocal IOL (PanOptix). Lawless et al. [17] reported that 15% of patients had dysphotopsia symptoms with the same trifocal IOL. However, Akman et al. [4] reported that dysphotopsia symptoms gradually decreased in all patients in consecutive postoperative examinations due to the neuroadaptation process.

Many studies have investigated factors associated with patient dissatisfaction after multifocal or trifocal IOL implantation. Most of these studies have focused on the relationship between the kappa (*κ*) angle and dysphotopsia [18–21]. In the most cited study, Prakash et al. reported that a higher angle *κ* indicates that a fovea-centric ray passes closer to the edge of the IOL rings, which causes halo and glare effects after multifocal IOL implantation [18].

Qi et al. chose cutoff values of 0.2 and 0.4 mm for the angle *κ* and reported incidence of higher glare and halo in groups with higher angle *κ* after trifocal IOL implantation [19]. At 3-month postoperative examinations, the incidence of halo was significantly higher (*P* < 0.05) in the groups with higher angle *κ* (13.8% in lower angle *κ* group, 0 ≤ *κ* ≤ 0.2; 24.2% in moderate angle *κ* group, 0.2 ≤ *κ* ≤ 0.4; and 51.8% in higher angle *κ* group, *κ* > 0.4). Moreover, the incidence of glare in those with higher angle *κ* group was significantly higher than in patients with lower values (*P* < 0.05). Unlike the results of our study, Qi et al. reported that the visual quality of patients decreased when angle *κ* was higher than 0.5 mm. Based on these studies, the incidence of dysphotopsia increases with angle *κ*.

However, we have not encountered a study investigating the relationship between dysphotopsia and angle *λ*, which is very similar to angle *κ*, as shown in Figure 1. As long as the fixation point is not close to the eye, these angles are nearly identical to each other [22–24]. Therefore, we measured the satisfaction of patients to determine the relationship between angle *λ* and dysphotopsia symptoms after trifocal IOL implantation. However, this study had two limitations. First, postoperative angle *λ* measurements were not available in our retrospective study. Second, the effect of the angle *κ* could not be evaluated as an independent variable since our clinic did not have the ability to measure it.

In conclusion, many patients report dysphotopsia symptoms that decrease patient satisfaction after trifocal IOL implantation. Preoperative examinations should be performed carefully to minimize the incidence of these symptoms. Patients with higher angle *λ* (≥0.5 mm) should be informed about possible dysphotopsia symptoms during nighttime activities until the completion of the neuroadaptation process.

## Figures and Tables

**Table 1:** Preoperative data.

	Mean ± SD		Range	*P* value
	*λ* < 0.5 mm	*λ* ≥ 0.5 mm		
UDVA (logMAR)	0.37 ± 0.28	0.38 ± 0.27	0.00, 1.00	0.825
Spherical equivalent (D)	0.71 ± 2.00	0.99 ± 2.28	−7.00, 5.375	0.486
CDVA (logMAR)	0.15 ± 0.21	0.13 ± 0.21	0.00, 0.90	0.562
Corneal astigmatism (D)	−0.51 ± 0.22	−0.49 ± 0.24	−0.75, 0.00	0.697
Ocular axis (mm)	23.39 ± 0.9	23.33 ± 0.97	20.79, 26.73	0.698
Anterior chamber depth (mm)	3.16 ± 0.38	3.10 ± 0.30	2.38, 3.86	0.355
IOL power (D)	21.27 ± 2.91	21.81 ± 2.38	11.5, 28.5	0.248
Scotopic pupil size (mm)	4.81 ± 0.82	4.82 ± 0.95	2.97, 6.97	0.936
Spherical aberration (*μ*m)	0.12 ± 0.07	0.13 ± 0.07	0.00, 0.30	0.506
High order aberration (*μ*m)	0.27 ± 0.11	0.31 ± 0.14	0.08, 0.80	0.071
Coma aberration (*μ*m)	0.17 ± 0.07	0.19 ± 0.10	0.00, 0.61	0.072
Angle *λ* (mm)	0.37 ± 0.08	0.80 ± 0.21	0.20, 1.40	<0.001
Target refraction^*∗*^ (D)	−0.03 ± 0.16	−0.02 ± 0.14	−0.49, 0.49	0.638

CDVA: corrected distance visual acuity; UDVA: uncorrected distance visual acuity. ^*∗*^Based on the SRKT formula.

**Table 2:** Postoperative patient parameters.

	Mean ± SD		Range	*P* value
	*λ* < 0.5 mm	*λ* ≥ 0.5 mm		
UDVA (5 m) (logMAR)	0.08 ± 0.06	0.09 ± 0.05	0.00, 0.20	0.406
UIVA (70 cm) (logMAR)	0.19 ± 0.09	0.21 ± 0.07	0.00, 0.40	0.355
UNVA (40 cm) (logMAR)	0.18 ± 0.10	0.17 ± 0.08	0.00, 0.40	0.450
Spherical equivalent (D)	−0.05 ± 0.34	−0.12 ± 0.35	−0.75, 0.00	0.284
Corneal astigmatism (D)	−0.37 ± 0.20	−0.43 ± 0.22	−0.75, 0.00	0.144

logMAR: logarithm of the minimum angle of resolution; UDVA: uncorrected distance visual acuity; UIVA: uncorrected intermediate visual acuity; UNVA: uncorrected near visual acuity.

## Data Availability

The data used to support the findings of this study are restricted by the Ethics Committee of Marmara University in order to protect patient privacy. Data are available from Dr. Sultan Kaya Ünsal (sultankayaunsal@gmail.com) for researchers who meet the criteria for access to confidential data.

## Additional Points

*What Was Known*. (i) Good visual acuity at all distances, positive refractive outcomes, and spectacle independence can be provided by trifocal IOL implantation. (ii) A large angle *κ* is one of the factors underlying patient dissatisfaction due to dysphotopsia after trifocal IOL implantation. *What This Paper Adds*. (i) The new trifocal IOL (Optiflex Trio) showed very good results for visual acuity at all distances, positive refractive outcomes, spectacle independence, and high patient satisfaction in daily life. (ii) A lambda (*λ*) angle greater than 0.5 mm may potentially cause dysphotopsia symptoms. (iii) Before surgery, patients with greater lambda angles (*λ* ≥ 0.5 mm) should be informed about possible dysphotopsia symptoms, especially during nighttime activities.
